# THPdb: Database of FDA-approved peptide and protein therapeutics

**DOI:** 10.1371/journal.pone.0181748

**Published:** 2017-07-31

**Authors:** Salman Sadullah Usmani, Gursimran Bedi, Jesse S. Samuel, Sandeep Singh, Sourav Kalra, Pawan Kumar, Anjuman Arora Ahuja, Meenu Sharma, Ankur Gautam, Gajendra P. S. Raghava

**Affiliations:** Bioinformatics Centre, CSIR-Institute of Microbial Technology, Chandigarh, India; Florida State University, UNITED STATES

## Abstract

THPdb (http://crdd.osdd.net/raghava/thpdb/) is a manually curated repository of Food and Drug Administration (FDA) approved therapeutic peptides and proteins. The information in THPdb has been compiled from 985 research publications, 70 patents and other resources like DrugBank. The current version of the database holds a total of 852 entries, providing comprehensive information on 239 US-FDA approved therapeutic peptides and proteins and their 380 drug variants. The information on each peptide and protein includes their sequences, chemical properties, composition, disease area, mode of activity, physical appearance, category or pharmacological class, pharmacodynamics, route of administration, toxicity, target of activity, etc. In addition, we have annotated the structure of most of the protein and peptides. A number of user-friendly tools have been integrated to facilitate easy browsing and data analysis. To assist scientific community, a web interface and mobile App have also been developed.

## Introduction

With the significant advancements in biologics and biopharmaceutical over the years, peptides and proteins have emerged with a host of new applications in the diagnostic as well as the therapeutic sector [1, 2]. As per the current calculations, the market for peptide and protein drugs is estimated around 10% of the entire pharmaceutical market and will make up an even larger proportion of the market in the future [3, 4]. Since early 1980s, a total 239 therapeutic proteins and peptides are approved for clinical use by US-FDA [5].

Since the introduction of first recombinant protein therapeutic, human insulin [6], proteins have emerged as a major new class of therapeutics with nearly 380-marketed pharmaceutical products. Proteins as therapeutics have drawn a lot of scientific attention because they are part of many receptors and channels in the membranes, which facilitate transport of molecules within the body [7]. Despite their involvement in various biochemical reactions, proteins also elicit high specificity with the molecular target [7]. On the other hand, peptides have also been used widely as therapeutics in cancer, diabetes, infectious diseases and many other disorders that have long been studied for a solution [8, 9]. An advantage of peptides over other drugs is that they are highly versatile, providing a wide variety of pharmaceutical targets, high specificity and show low toxicity levels [5, 9]. A total 60 peptide-based drugs are already in the market and several other therapeutic peptides are currently being evaluated in different phases of clinical trials [5].

Despite the several advantages, there are a few drawbacks associated with these protein and peptide-based therapeutics. Limitations like low solubility, proteolytic degradation, relatively short circulating half-life, physiochemical instability and immunogenicity restrict their ease of administration and compliance at the user end [1, 4, 10]. They generally have high molecular weights, low lipophilicity and presence of some charged functional groups, which tend to hamper their absorption at various sites of the gastro-intestinal tract (GIT), resulting in their low bioavailability [4]. Several strategies like glycosylation, PEGylation, manipulation of amino acid sequence, conjugation to serum albumin have emerged in order to improve the pharmacokinetic and pharmacodynamics properties as well as to decrease the immunogenicity and proteolytic cleavage [1, 5]. With the advent of new technologies, incorporation of non-natural amino acids and pseudo peptide bonds also provide wider chemical diversity with diversified potentials and making it economically more viable [4, 11].

Over the last decade, a plethora of databases encompassing information on protein and peptides with different therapeutic functionalities [12–19] have been developed, reflecting the increased interest for proteins and peptides as therapeutics among the scientific community. A substantial interest has also been shown towards peptide-based subunit vaccine and immunotherapeutic [20]. The information on US-FDA approved protein and peptide therapeutics along with their pharmacokinetic / pharmacodynamic properties, their advantages, chemical modifications, and their limitations are very important. However, this information is not easily accessible and it is scattered in the literature. To the best of authors’ knowledge, to date, no single freely available platform exists which is totally dedicated to US-FDA approved protein and peptide therapeutics. Therefore, keeping the above facts in mind, we have developed THPdb, which is a comprehensive resource of all US-FDA approved protein and peptide therapeutics along with their corresponding drug variants available in the market. We anticipate that the information available in the THPdb will certainly be very helpful to the researchers working in the field of peptide and protein-based drug discovery.

## Methods and materials

### Data collection and compilation

In order to collect and compile the latest information on US-FDA approved protein and peptide therapeutics; first, we searched DrugBank [21] using keyword “biotech drugs” with “Approved” and “Investigational” filters. This search resulted into 369 biotechnology-based drugs on 20^th^ Apr 2017. Among these, only 239 were peptides and proteins, which were subsequently shortlisted for inclusion in the THPdb. Further, in order to provide the complete profile and comprehensive information (regarding the mechanism of action, physical form, route of administration, category of activity, interaction with other drugs, recommended dosages, contraindications, side effects and the explored therapeutic area of these shortlisted peptides and proteins), specific searches were carried out in the PubMed and patent databases using strings ‘therapeutic proteins’, ‘therapeutic peptides’, and using the name of the individual protein and peptide. These searches resulted into a total 985 publications and 70 patents. The relevant information under various heads was collected and compiled manually from these articles.

In order to improve the pharmacokinetic parameters like the volume of distribution (Vd), clearance (CL), or the bioavailability (AUC), different modifications are being made to the existing drug molecules (drug variants). Here, in THPdb, each such modification with their brand names compiled in a separate entry. Therefore, multiple entries have been made if a drug molecule has been modified in different ways or it is being sold under different Brand names. Finally, a total 380 drug variants of these peptides and proteins have been systematically compiled in THPdb.

### Database architecture and web interface

**Fig 1:**
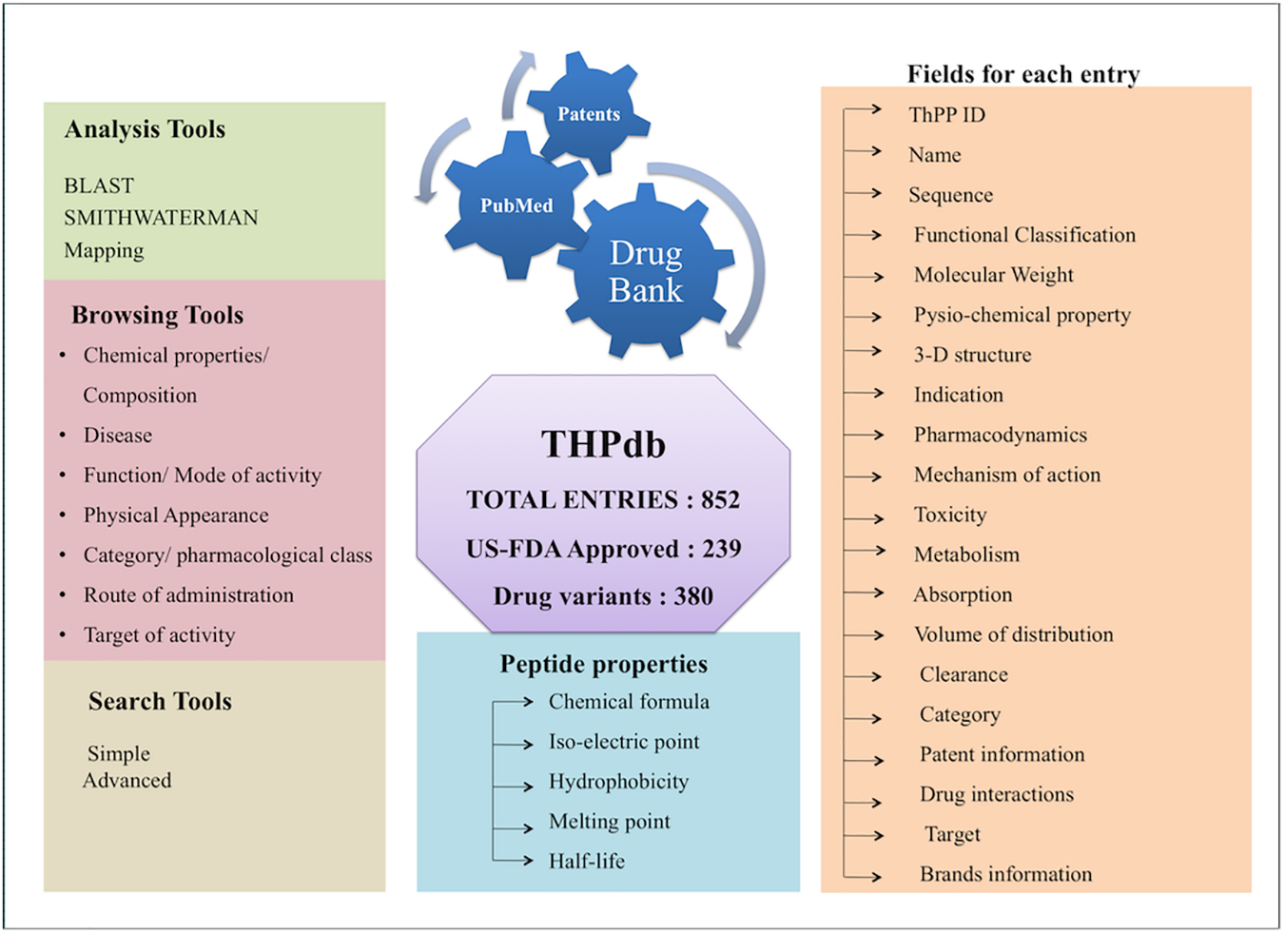
Schematic representation of THPdb architecture.

THPdb database has been built using a standard platform based on the Linux-Apache-MySQL-PHP (LAMP). Red Hat Linux (version 6.2) as the operating system, MySQL (version 14.12) for managing the data and Apache (version 2.2.17) as the HTTP server was used for developing this database. HTML5, PHP, JAVA scripts have been used for developing the mobile and tablet compatible front ends and MySQL for developing the back end. For the entire database interface and the common gateway, networks PHP and PERL coding have been used. The architecture of THPdb database is given in Fig 1.

### Organization of data

#### Primary information

Information on protein and peptide therapeutics were obtained manually from different resources and stored as primary information. Each entry includes the following information: name of the therapeutic molecule, amino acid sequence or primary structure, molecular weight, chemical formula, half-life, melting point, hydrophobicity, isoelectric point, toxicity, metabolism, pharmacodynamics, mechanism of action, toxicity, indication/disease, metabolism, absorption, Vd, CL, categories, patent no.’s, date of issue and date of expiry of the patent, drug interactions and the targets. In addition, information of company name, brand name, formulation, recommended dosage, side effects, contraindications, and the route of administration have also been collected and stored as the primary data.

#### Derived information

**Fig 2:**
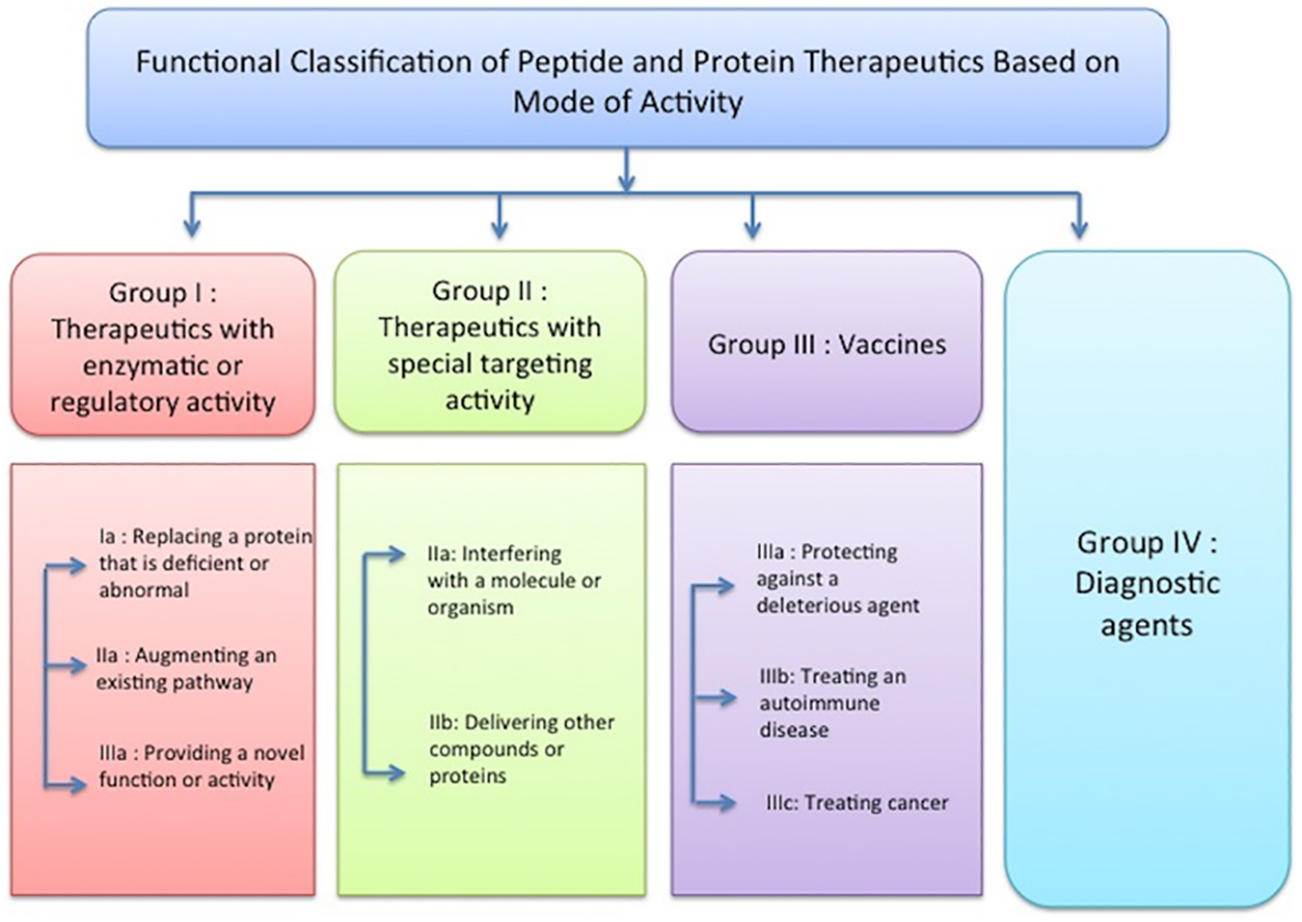
Overview of the function/mode of activity based classification of therapeutic proteins and peptides.

Apart from the primary information, information related to function/ activity of the peptides and proteins have also been compiled. Based on the role or mode of activity, therapeutic peptides and proteins are classified into four groups [7], Group I consist of the proteins with enzymatic or regulatory activity, Group II of those with specific targeting activity, Group III are protein vaccines, and Group IV consists of diagnostic agents. The proteins with enzymatic or regulatory activity are further sub-divided into three categories: (Ia) those replacing a deficient or abnormal protein, (Ib) those augmenting an existing pathway, and (Ic) those providing a novel function or activity. Therapeutics with specific targeting activity being sub-divided into two categories: (IIa) peptides interfering with a molecule or organism, and (IIb) peptides delivering other compounds or proteins. Similarly, group III contains protein vaccines, which are sub-classified as (IIIa) vaccines, which protect against a deleterious foreign agent, (IIIb) which treats autoimmune diseases and (IIIc) treats cancer. The last category corresponds to proteins that can be used as diagnostic agents. This classification scheme is explained previously [7]. This information has been stored as secondary data (Fig 2).

To understand the nature of these therapeutic peptides and proteins, it is necessary to understand their physicochemical properties, which has been calculated by in-house PERL scripts that were used to compute amino acid composition and frequencies. All these properties are helpful in analyzing the trends and determining the amino acids dominating in such proteins and peptides. This physiochemical property based information is also stored as secondary data in the database for browsing of peptides.

Many properties exhibited by the therapeutic peptides and proteins are due to their structural characteristics. Thus, it is important to understand the relation between the structures of the biomolecules and its function. We have thus provided the structural annotation of each protein and peptide in our database using the following approaches. The structural information was first extracted from the DrugBank using the Protein Data Bank (PDB) entries [22] (if present) given in the DrugBank followed by downloading it from PDB. In cases, where no structural information was available in either DrugBank or PDB, we used bioinformatics tools for structure prediction. I-TASSER software [23] was used for predicting structure of proteins having more than 30 amino acids. In case, length of a peptide is 30 or less, then PEPstrMOD [2], which is updated version of PEPstr [24], has been used. If a protein or peptide has any modified residue (e.g. non-natural residues and capping of terminal residues), it was predicted using PEPstrMOD containing special force field libraries to handle modified residues.

### Implementation of tools

#### Data retrieval or search tools

This module of THPdb has been designed to facilitate searching the data using simple and advanced search options. In simple search module, the user can submit the query against any field of the database such as name, sequence, category, classification based on their role/mode of activity, isoelectric point, indication etc. This option results into a customized output according to the search query. In the advanced search module, the user can submit multiple queries simultaneously with Boolean expressions (eg. AND, OR and NOT). Along with these tools and data, we have included a unique feature in our database, which are the downloadable self-explanatory power-point presentations. These presentations are freely available and can be downloaded. The user can use these for academic and research purposes.

#### Browsing

A user-friendly browsing interface has been developed to facilitate easy retrieval of the information from THPdb. We have stored physicochemical properties of each therapeutic peptide and protein such as the hydrophobicity, isoelectric point, melting point, molecular weight and half-life. Users can browse peptide and protein entries for the desired physicochemical properties.

To access the data in THPdb, various cross-linked browsable tables have been provided. The users can browse on seven major fields: (i) disease area of activity, (ii) number of amino acids in the protein or peptide, (iii) classification based on their function/mode of activity, (iv) physical appearance state (v) route of administration (vi) category/pharmacological class and (vii) target based classification.

#### Sequence alignment

In order to facilitate sequence similarity based search, we have integrated BLAST and Smith-Waterman algorithm. Users can submit their protein or peptide in FASTA format with default or desired parameters of BLAST [25]. The server performs BLAST search for the user’s query sequence against the amino acid sequences or the primary structures of all the peptides and proteins in the database. Similarly, Smith–Waterman algorithm [26] has also been integrated. In addition to above similarity search facility, the server also allows sequence mapping based on identical residues. Sub-search and super-search have been integrated as two mapping options. By using sub-search module, user can map query peptide against all the peptides and proteins entered in THPdb, whereas super-search allows mapping of the protein sequence against THPdb and identification of segments that are identical to therapeutic peptides.

### Mobile App

To assist the mobile users, we have devloped a mobile App. THPdb (mobile app) is a comprehensive database application of US-FDA approved peptide and protein therapeutics developed to facilitate its smooth usage on mobile platforms. The current mobile version of THPdb compatible with android mobiles exploits python(v2.7.11) and kivy(v1.9.2). The App lists various disease types and a drop down menu of potential therapeutic peptides followed by detailed information of drugs for individual protein and peptide. The important information for individual entity has been sorted into individual panels called “peptide-card” which can be easily downloaded. Each peptide-card features/hosts information about the peptide/ protein therapeutics, their description, sequence, indication, pharmacodynamics, mechanism of action, ADME information, toxicological information, metabolism, absorption, half life, clearance rate, patent information, drug-drug interactions, potential targets, physicochemical properties to name a few. The ‘THPdb’ mobile application is provided in standard “.apk” file format which can be downloaded from http://crdd.osdd.net/raghava/thpdb/mobile.php

#### App working outline

**Fig 3:**
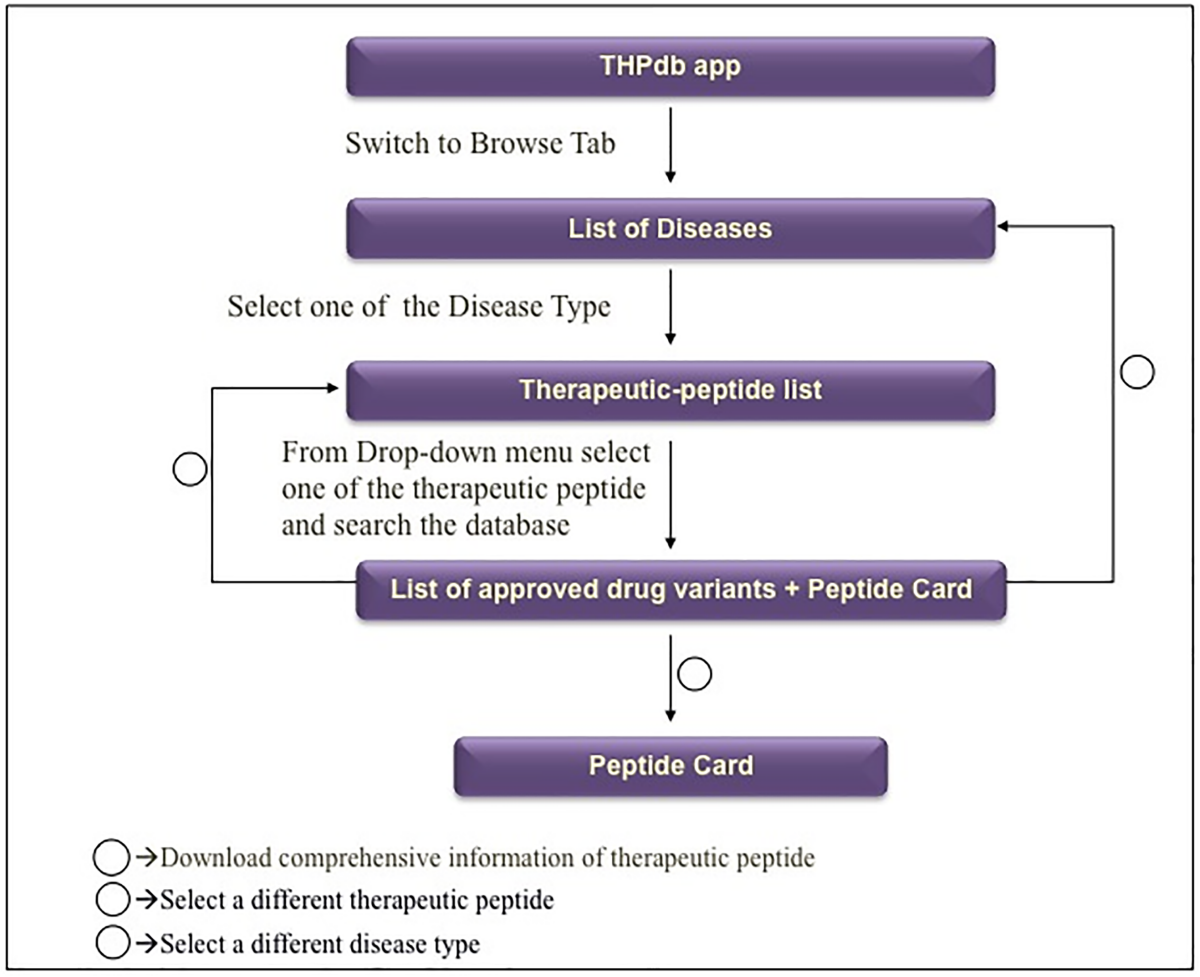
The flow diagram explains the work and design of THPdb mobile application.

THPdb app is divided into various levels to provide an interactive outline of the database. As shown in Fig 3, database exploration begins with the selection of a disease type from the “Browse” tab. In the next level, a peptide of interest is searched among the list of US-FDA approved peptide and protein therapeutics for the selected diseases to obtain a list of potentially approved drug(s). The hit shares a handful of information like drug brands, their manufacturers, and route of administration of corresponding therapeutic peptide /protein. More comprehensive information of important fields can be retrieved by downloading the “Peptide Card” that is available at the bottom of the each peptide page.

## Results

### THPdb data statistics

The current version of THPdb holds a total of 852 entries providing information on 239 US-FDA approved peptide and protein drugs and their 380 drug variants. Corresponding drug variants of therapeutic proteins and peptides were classified on the basis of a classification presented by Leader et al. Of the 380 drug variants, 229 involved in regulatory and enzymatic activity followed by 78 belonging to the therapeutics with special targeting activity. A total of 58 drug variants belong to the vaccines category and 15 to the diagnostic agents.

In addition, these drug variants have also been grouped on the basis of disease in which they are being used for therapy. A total of 89 drug variants show activity in case of metabolic disorders, 80 have activity in the immunological disease area, 74 for hematological diseases, 61 in the cancer therapy, 63 in hormonal disorders, 46 variants useful for genetic disorders, 35 in infectious disease, 14 in cardiovascular disorders, 10 have the potential to cure bone disorders, 07 used in neurological disorder, 06 for respiratory disorder, 05 variants are given as adjunct, 03 in eye disorder, and 01 variant has been used in malabsorption disorder.

**Fig 4:**
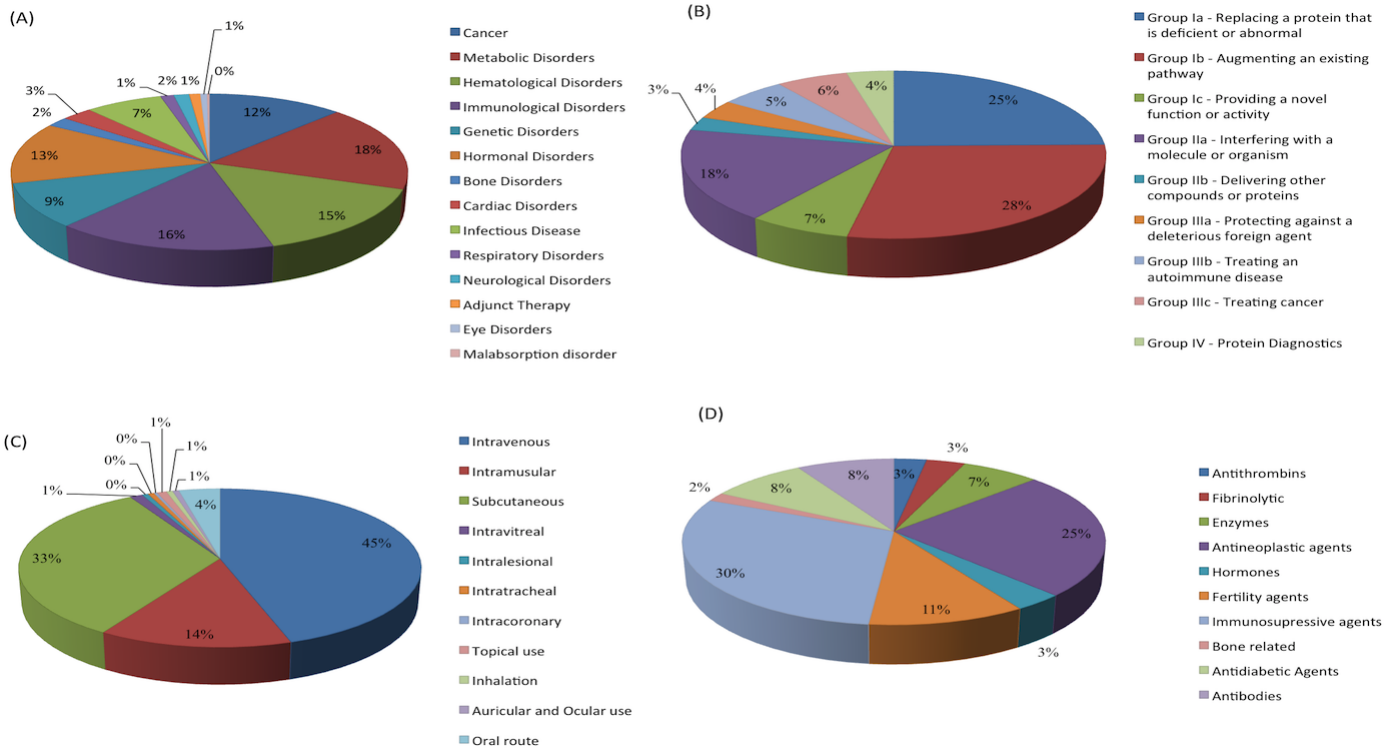
A schematic representation of distribution of therapeutic peptides and proteins based on disease, function/ mode of activity, route of administration, and pharmacological class.

The routes by which these variants are being delivered have also been compiled systematically. Total 158 drugs by intravenous infusions, 116 drug variants are delivered by subcutaneous injections, 49 drugs by intramuscular route, 13 drugs via the oral route, 4 by intra-vitreal, 2 by intralesional, 2 by intratracheal, 1 by intracoronary, and 3 drug variants are being used externally as ointments. All these data statistics have been summarized in Fig 4.

In order to assist users, we have provided power-point presentations containing detailed information of each US-FDA approved protein and peptide therapeutic including their sequence or primary structure, molecular weight, chemical formula, description, indication, pharmacodynamics, mechanism of action and its interaction with other drugs. These presentations can be downloaded from the THPdb website.

### Comparison with existing resources

Despite the fact that peptides and proteins are gaining tremendous scientific attention as therapeutics nowadays, no single repository exists till date which can provide comprehensive information on all US-FDA approved protein and peptide therapeutics. However, two databases, ChEMBL [27] and DrugBank provide the information on US FDA approved protein and peptide therapeutics to a certain extent but they don’t cover all the US-FDA approved protein and peptide therapeutics and their drug variants. As of 1st May 2017, ChEMBL provides information of only 191 protein and peptide therapeutics and information of 48 protein and peptide therapeutics is missing. In addition, among these 191 protein and peptide therapeutics, ChEMBL, provides structural information of only 148 protein and peptide therapeutics. Similarly, in case of drug variants, ChEMBL provides information of 144 variants only. DrugBank store some information about protein and peptide therapeutics, but it doesn’t give detailed description of corresponding drug variants of these peptide and protein therapeutics. In addition, DrugBank provides PDB ID of only 107 protein and peptide therapeutics, whereas THPdb provide structural information of about 156 protein and peptide therapeutics. In order to overcome the limitations of the above resources, we have incorporated detailed information of all 239 protein and peptide therapeutics and their 380 variants. Besides this, comprehensive information (their sequence, physico-chemical properties, structure, pharmacokinetics, pharmacodynamics properties, etc) of each protein and peptide therapeutic and their drug variant was provided in the form of power-point presentations. User can freely download the power-point presentations. Thus, THPdb provides the latest and comprehensive information and is a complementary resource to the existing databases.

## Discussion

Small peptides and proteins were never considered as the ideal therapeutic molecules, however, over the last few decades, a revival of interest in peptide and protein therapeutics has been seen among the pharmaceutical and biotech industries [1]. This can be evidenced by the increasing rate of peptides and proteins entry per year in the clinical trials [9]. In 2012 alone, six peptides were approved as drugs by US-FDA [11]. Despite the immense popularity of proteins and peptides as therapeutics, the information of all already US-FDA approved protein and peptide therapeutics is far from reach. Therefore, in the present study, an attempt has been made to provide a comprehensive insight on the US-FDA approved peptide and protein therapeutics including all the information on their physical, chemical and activity related characteristics. In THPdb, all this information has been compiled in a systematic manner, which will help the researchers to look into a variety of aspects of a particular protein or peptide simultaneously.

Development of a drug is a complex and time-consuming process. Even though peptides/proteins are highly active in their native form, they need to be modified in several ways in order to become a drug molecule. These series of changes make the lead molecule as a better therapeutic candidate and the prior knowledge of such changes or modifications will be very useful in developing future drugs. The peptide researchers may be interested to know the half-life, chemical modifications, immunogenicity, solubility, side effects, toxicity, pharmacodynamics, different formulations, dosage forms, and the different brand names under which these drugs are currently sold in the market. In this context, THPdb is very helpful in the development of novel therapeutic entities, particularly during the initial phases of drug discovery by understanding how the drawbacks have been overcome in US-FDA approved peptide and protein therapeutics. For example, the various modifications made to improve the therapeutic profiles of US-FDA approved proteins will give researchers many clues to improve the therapeutic profile of their lead molecules.

### Utility of database

One of the major advantages of THPdb is that users can get the complete information on US-FDA approved protein and peptide therapeutics, which was not previously available on a single platform. THPdb provides its users with numerous services, like (i) users can check whether their peptides or proteins of interest has already been reported or not, and how different or similar their protein or peptide is with the existing approved therapeutics, (ii) what kind of modifications has been done to increase therapeutic competency of the therapeutics like half-life, rate of clearance, etc., and (iii) users can exploit the structural information of these peptides provided in THPdb for molecular dynamics or docking studies. In addition, a mobile application is also developed with the aim of making it easy for the researchers.

Besides this, THPdb also provides self-explanatory resource in power-point format, which will be very helpful for academicians, and researchers and we hope that it will accomplish our main aim behind this database *i*.*e* “academic enhancement” and “knowledge enrichment”.

#### Case study: How to retrieve information about “Insulin” from THPdb

**Fig 5:**
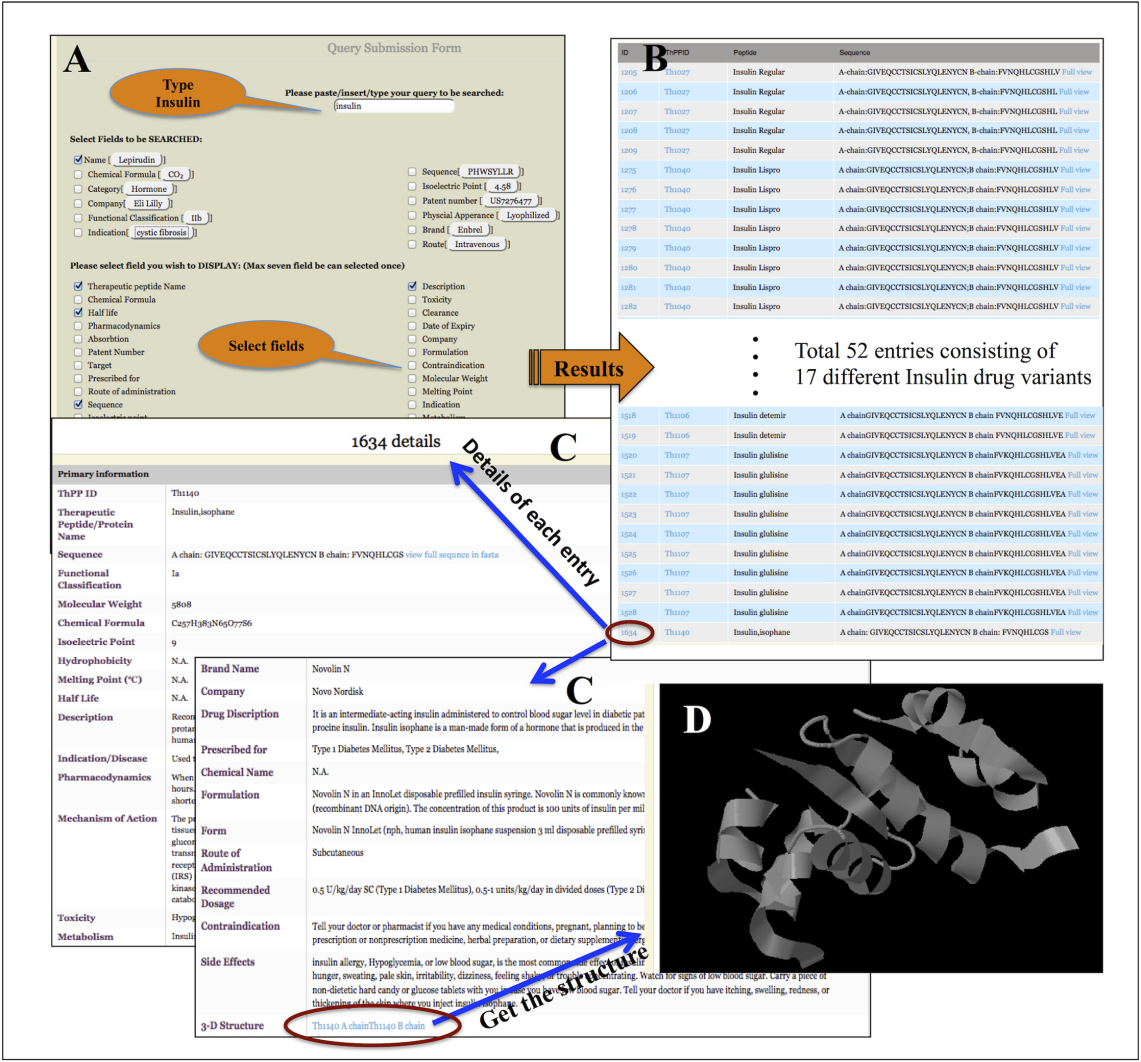
Representative screen shots of THPdb demonstrating the flow of information after submission of a query in simple search page.

Here we have shown step-by-step about what kind of information a user can fetch from THPdb. We have use Insulin as therapeutics in a case study to explain this. Below is the description:

If user wants to know about the different type of insulin drugs (drug variants) approved by US-FDA, user has to type Insulin in the space provided in the simple search option (Fig 5A) or alternatively user can use advance search tool as well. On simple search page, a number of fields are provided on which user can fetch the information. At a time, user can select a maximum of 7 fields to be displayed. After selecting fields, user has to submit the query and search will end up with all insulin-based drugs and its variants available in the market under different brand names. For example, here, a total 52 entries comprising 17 insulin drugs and its variants are displayed along with information on selected fields (Fig 5B). User can also click on individual entry ID in order to obtain the detailed information for a particular insulin drug as shown in Fig 5C. By doing so, user can get the all possible information of that particular drug like sequence, physico-chemical properties, pharmacokinetics and pharmacodynamics, half-life, side-effects, route of administration, dosage form, brand name, etc. In addition, user can also get the structural information by clicking on 3-D structure option provided in the detailed information page Fig 5D.

### Limitations and database update

While compiling the data under various categories for each peptide and protein, we faced some challenges, as the sequence of 57 of the approved peptides and proteins could not be obtained from any of the available sources. These are mainly the monoclonal antibodies and a few peptide antibiotics. This information will be updated in the database as soon as the new information is available in the literature.
